# Mindfulness-Based Symptom and Stress Management Apps for Adults With Chronic Lung Disease: Systematic Search in App Stores

**DOI:** 10.2196/mhealth.9831

**Published:** 2018-05-15

**Authors:** Otis L Owens, Jenay M Beer, Ligia I Reyes, David G Gallerani, Amanda R Myhren-Bennett, Karen K McDonnell

**Affiliations:** ^1^ College of Social Work University of South Carolina Columbia, SC United States; ^2^ College of Public Health University of Georgia Athens, GA United States; ^3^ School of Social Work University of Georgia Athens, GA United States; ^4^ Department of Health Promotion Education and Behavior University of South Carolina Columbia, SC United States; ^5^ College of Nursing University of South Carolina Columbia, SC United States

**Keywords:** mindfulness, lung neoplasms, chronic obstructive pulmonary disease, mobile apps, review

## Abstract

**Background:**

Up to 70% of lung cancer survivors are affected by chronic obstructive pulmonary disease (COPD), a common, debilitating, comorbid disease. Lung cancer and COPD are both characterized by symptoms such as breathlessness, fatigue, and psychological distress. These distressing chronic symptoms are exacerbated by stress and detract from an individual’s quality of life.

**Objective:**

The aim of this study was to identify and evaluate evidence-based, commercially available apps for promoting mindfulness-based strategies among adults with a COPD or lung cancer history (ie, chronic lung disease).

**Methods:**

For this review, an interdisciplinary research team used 19 keyword combinations in the search engines of Google and iOS app stores in May 2017. Evaluations were conducted on the apps’ (1) content, (2) usability heuristics, (3) grade-level readability, and (4) cultural sensitivity.

**Results:**

The search resulted in 768 apps (508 in iOS and 260 in Google stores). A total of 9 apps met the inclusion criteria and received further evaluation. Only 1 app had below an eighth-grade reading level; the ninth one did not have enough text to calculate a readability score. None of the 9 apps met the cultural sensitivity evaluation criteria.

**Conclusions:**

This systematic review identified critical design flaws that may affect the ease of using the apps in this study. Few mobile apps promote mindfulness-based strategies among adults with chronic lung disease (ie, COPD or lung cancer or both), but those that exist, overall, do not meet the latest scientific evidence. Recommendations include more stringent regulation of health-related apps, use of evidence-based frameworks and participatory design processes, following evidence-based usability practices, use of culturally sensitive language and images, and ensuring that content is written in plain language.

## Introduction

**Prevalence of Lung Cancer and Chronic Obstructive Pulmonary Disease**

Lung cancer is the second most commonly diagnosed cancer in the United States, accounting for 13.2% of new cases in 2017 [1]. It is expected that 222,000 new lung cancer cases will be diagnosed in 2017 [1], adding to the more than 500,000 individuals currently living with the disease. Chronic obstructive pulmonary disease (COPD) is also a common, debilitating disease characterized by breathlessness and fatigue, which affects almost 15 million individuals and is the third leading cause of death [2,3]. Several studies demonstrate strong links between lung cancer and COPD, such as their common environmental, genetic, and epigenetic risk factors and their similar pathogenic mechanisms for activation [4-6]. In particular, those with COPD are 5 times more likely to develop lung cancer than those individuals without the disease [7]. These links between lung cancer and COPD, in part, explain why up to 70% of lung cancer survivors are affected by COPD and many of the survivors of both diseases describe similar distressing symptoms that negatively affect their daily lives [3,8,9]. Therefore, some researchers are proposing additional studies that not only further elucidate the link between COPD and lung cancer (ie, chronic lung disease) but also generate therapies that can be used to alter the mechanisms involved in both disease processes [4,6].

**Mindfulness-Based Stress Reduction for Chronic Lung Disease**

The 5-year survival rates have steadily improved over the past decade for chronic lung disease; however, these rates are highly dependent on the stage of disease at the time of diagnosis [10-12]. Efforts to decrease the burden of chronic lung disease (ie, early detection, improved treatments, symptom management, more accessible smoking cessation strategies) will likely lead to a larger population of longer-term cancer survivors [8,9,13]. The American College of Chest Physicians has made recommendations regarding complementary therapy modalities that may improve the quality of life for survivors of chronic lung disease. These therapies are inclusive of mindfulness-based stress reduction (MBSR) strategies such as meditation or yoga, which in turn have shown to relieve symptoms related to chronic lung disease [14,15]. Although MBSR is generally administered in-person, available technologies provide many opportunities for dissemination of these therapies in other ways, to fit individual schedules and needs.

With the growing ubiquity of mobile technologies, survivors are increasingly using the Internet as a resource for health information [16]. According to the Pew Research Center, 72% of Internet users say they have searched online for health information [17]. Another recent national study suggests more than half of mobile phone users have downloaded a health-related mobile app (henceforth referred to as app) [18]. These users were also found to place high trust in these apps’ accuracy and experienced positive health effects [18]. Despite the frequency and use of these technologies, there is no regulatory authority to validate the legitimacy of health-related content published through these commercial apps, nor is there a mechanism to enforce standards to ensure that the information is accessible by diverse populations [19].

### Objective

Besides Coulon et al’s [20] review of stress management apps, there have been no systematic reviews that focused on the evaluation of MBSR apps for individuals with chronic lung disease. Therefore, the objective of our review was to identify and evaluate apps available in the Google Play Store (Android devices) and/or the Apple Store (for iOS-based devices) for promoting mindfulness-based strategies specifically among adults with chronic lung disease. The primary aim was to evaluate whether and to what extent the content of these apps is evidence-based and transparent in its purpose, development, and content (eg, provides contact information for its developers). The secondary aim was to evaluate the usability, readability, and cultural sensitivity of these apps. Our ultimate goal is to determine if these apps can improve the quality of life among racially and ethnically diverse populations of lung disease survivors. Furthermore, we want to make recommendations to improve these apps, so that health information can be more accessible, accurate, and effective for these populations.

## Methods

### Keywords and App Search

A total of 19 keyword combinations were created using COPD or Lung Cancer, followed by MSBR, meditation, breathing, diaphragmatic, stress management, progressive muscle relaxation, or yoga. Each combination was searched in Google and iOS app stores in May 2017 using compatible mobile devices (see Textbox 1).

### App Review Overview

**Figure 1:**
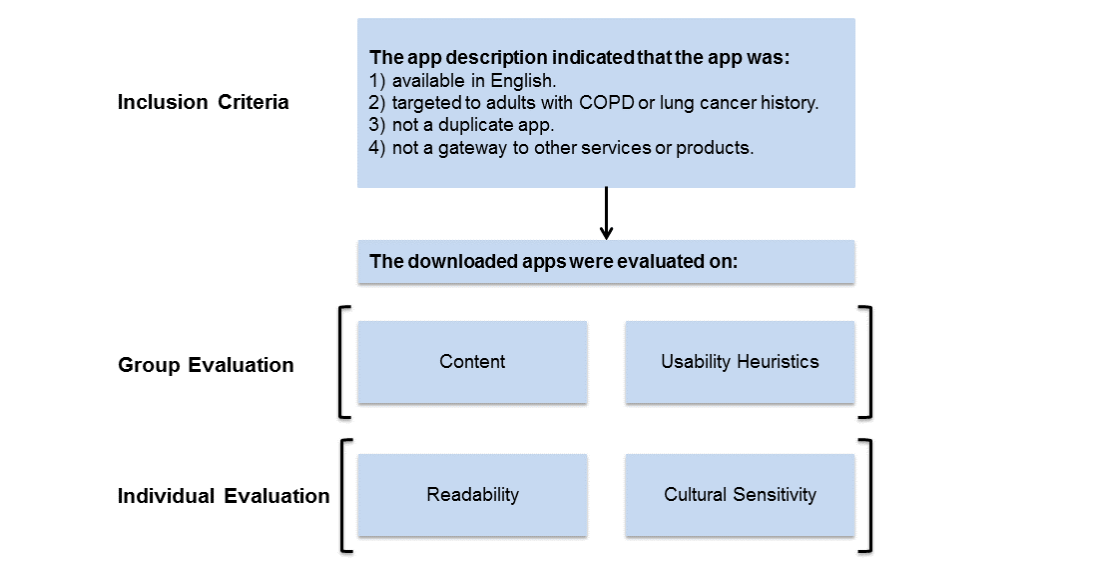
App review steps. COPD: chronic obstructive pulmonary disease.

We adapted existing procedures by Coulon and colleagues [20]. We evaluated the apps for content, usability heuristics, readability, and cultural sensitivity. Evaluation was conducted in a multidisciplinary group setting involving 5 to 6 reviewers (ie, group evaluation) for content and usability. Readability and cultural sensitivity was conducted independently by 1 and 2 reviewers, respectively (ie, individual evaluation)*.* Drawing on the work of Coulon and colleagues [20], we used 1 level of inclusion criteria. We used app descriptions to determine whether an app met 1 of the following 4 criteria: (1) available in English, (2) targeted adults with chronic lung disease, (3) was not a duplicate within or across app stores, and (4) was not a service gateway, such as required subscriptions beyond the app. The apps were downloaded for further investigation if the 4 criteria could not be determined from the description (Figure 1).

### Inclusion Criteria

**Figure 2:**
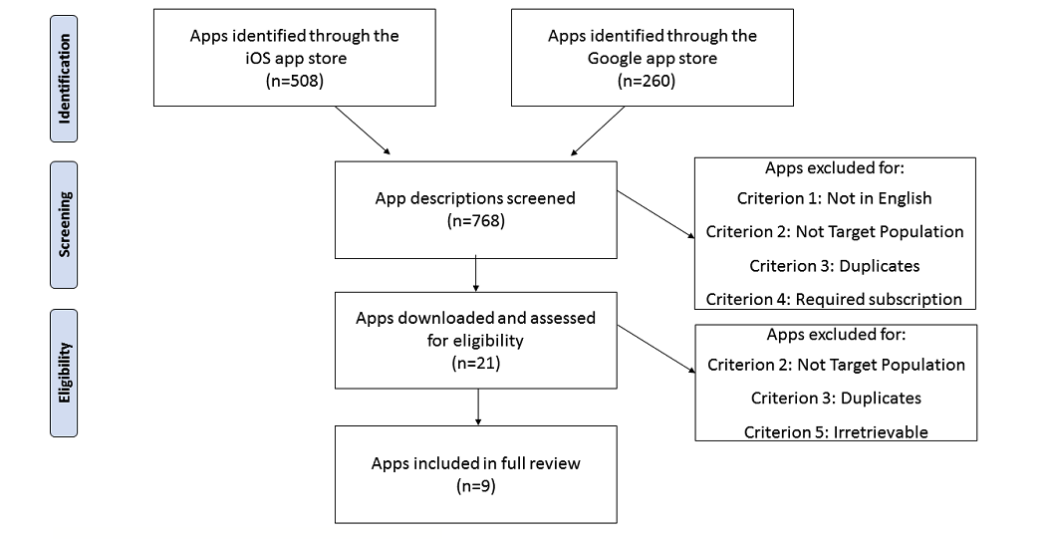
App inclusion flow.

The 19 keyword combination searches yielded 768 apps—508 apps in the Apple Store and 260 in the Google Play Store. Figure 2 illustrates the procedure for determining if apps met the inclusion criteria. Of these, 37 apps were not in English (criterion 1). Of the remaining 731 apps, 639 did not target adults with chronic lung disease (criterion 2). This left 92 apps, of which 69 were duplicates of each other (ie, failed criterion 3), and 2 required additional subscriptions or purchases (thereby failing criterion 4). Thus, after the inclusion assessment, 21 apps remained. Upon downloading these apps, 4 failed to meet criterion 2 (brief or generic descriptions previously prevented this determination). Moreover, 3 additional apps failed to meet criterion 3 (also because the nature of the content had been obscured until downloading). Of the remaining 14 apps, 5 were irretrievable or malfunctioned during download (ie, failed criterion 5). In total, 9 apps were selected for further evaluation. None of the 9 remaining apps were duplicated across both app stores. In addition, all apps were free, though 1 app had the option to purchase additional breathing exercises.

**App review keywords. COPD: chronic obstructive pulmonary disease; MBSR: mindfulness-based stress reduction.**

- COPD breath
- COPD MBSR
- COPD meditation
- COPD mindfulness-based stress reduction
- COPD stress breathing
- COPD stress diaphragmatic breathing
- COPD stress management
- COPD stress progressive muscle relaxation
- COPD yoga
- Lung cancer breath
- Lung cancer MBSR
- Lung cancer meditation
- Lung cancer mindfulness-based stress reduction
- Lung cancer stress breathing
- Lung cancer stress diaphragmatic breathing
- Lung cancer stress management

### Content Evaluation

We established 4 content evaluation domains: (1) evidence of science, (2) scientific strategy and engagement, (3) evidence-based stress management, and (4) transparency in its purpose, development, and content. Although all of our criteria were adapted from Coulon et al’s study, we included 2 of their inclusion criteria as evaluation criteria [20] (see Textbox 2 for domain definitions and their respective criteria). Across the 4 domains, we developed a coding sheet (converted to a Google Form) to determine whether criteria were met, somewhat met, or not met.

The multidisciplinary research team of 5 to 6 reviewers met 5 times to conduct these evaluations in a group setting. We operationalized this evaluation by downloading a given app and projecting it onto a big screen to familiarize ourselves with the app content (for 10-20 min). One reviewer was responsible for navigating through the app. Some discussion ensued if reviewers had uncertainties or questions about a given app that warranted additional explanation. Each researcher then independently scored the content in Google Forms guided by the criteria in Textbox 2. The response options for each of these criteria were as follows: met, somewhat met, or did not meet. Reviewers were also provided with a space to justify their given response. The content evaluation took 20-45 min. All results (including comments) were concatenated using Google Forms, and these results are reported based on majority consensus.

### Usability Heuristics Evaluation

An expert in human-computer interaction prepared the usability heuristics evaluation questionnaire based on Nielsen’s 10 usability heuristics (see Table 1) [21-23]. The questionnaire was administered via Google Forms. The review team, similar to the process described above for the content evaluation, evaluated usability heuristics of each app as a group and individually assigned a numerical value to rate the severity of each heuristic violation (see Table 1 for the heuristics on which each app was evaluated, accompanied by questions and response options. In addition, similar to our content evaluation, results were determined based on majority consensus).

### Grade-Level Readability

Readability was evaluated using the approach employed by Smith and colleagues [20]. Readability was measured using Readibility.io (computer software by Added Bytes, Sussex, England). This software provides grade-level scores according to 5 standardized reading scales (Flesch-Kincaid Grade Level, Gunning Fog Score, Coleman-Liau Index, SMOG Index, and Automated Readability Index) [24] along with an average of the 5 scores. We retrieved the average score using 125 to 150 words of text from each app.

### Cultural Sensitivity

To our knowledge, there are no validated measures for evaluating the cultural sensitivity of commercially available apps. Therefore, we adapted the Cultural Sensitivity Checklist (CSC) developed by Friedman and Hoffman-Goetz [25] to evaluate our material for cultural sensitivity for African Americans. We are interested in the apps’ sensitivity among African Americans specifically because they have higher rates of lung cancer [26] and earlier onsets of COPD [27]. The CSC checklist was designed to evaluate printed material, but has also been used for online material [28]. The original checklist contains 8 items, of which only 5 were pertinent to our study. (Two items overlapped with our content and readability evaluations and 1 focused on cancer prevention instead of symptom management.) We scored each app on the basis of whether it met, somewhat met, or did not meet the CSC criteria. To establish intercoder reliability, 2 reviewers conducted separate evaluations for each app. Percent agreement was calculated by dividing the total number of agreements by the total possible items. The 2 reviewers reached 100% agreement.

**Domains and criteria of content evaluation.**

**Domain 1: evidence of science**

- App contains terminology (or other form of) evidence, research, science, and/or study
- App contains scientific reference for the app strategy
- App contains evidence that it was developed by an established institution that conducts research

**Domain 2: scientific strategy and engagement**

- App contains a skill-building, behavior-change component as evidenced by skill-related instructions
- App provides available opportunities for continued engagement

**Domain 3: evidence-based stress management strategies and structures**

- Strategies
  - *Diaphragmatic breathing* refers to slow, paced breathing in through the nose and out through the mouth by contracting the diaphragm or distending the abdomen; monitoring muscle tension while tensing and releasing muscles sequentially throughout the body
  - *Meditation, mindfulness* refers to intentional focus on thoughts and sensations experienced in the present moment, without judging them positively or negatively
  - *Cognitive restructuring* refers to the identification, evaluation, monitoring, and altering specific thoughts that may be distorted, unhelpful, or maladaptive
  - *Active coping, behavioral activation* refers to goal setting and engagement in activities that may improve mood and a sense of wellness, with the purpose of preventing or decreasing avoidant and isolative behaviors that can occur in times of duress
  - *Seeking social support* refers to engaging with trusted others who may provide emotional or functional supports (eg, calling a friend at a time of distress)
  - Problem solving refers to an attempt to remove a stressor, or to reduce its magnitude, frequency, or duration, by describing the problem, brainstorming solutions, selecting and testing a solution, and refining the solution
  - *Visualization, imagery* refers to the use of the 5 senses to imagine a specific stimulus (eg, a place or thing) in great detail, to achieve a state of relaxation, pleasure, or comfort

- Structures
  - *Assessment:* App provides an opportunity to complete a measure of perceived stress
  - *Self-monitoring:* App provides ongoing opportunities to rate perceived stress and/or behavioral indicators of evidence-based stress management
  - *Psychoeducation:* App provides educational information on the benefits of evidence-based stress management strategies and/or mechanisms of action

**Domain 4: transparent app presentation**

- *Authoritative:* App should state the qualifications of the app authors or developers; states degrees and/or specific training should be present
- *Complementary:* App should state that the app content should support, not replace, medical care and provider-patient relationships
- *Confidentiality or privacy*: App should state the privacy and confidentiality securities for personal data submitted to the site by the user
- *References:* App should state the source(s) of published information
- *Justification:* App should state the content of published information that supports claims relating to benefits and performance
- *Contact details:* App should provide information for contacting developers or app managers
- *Financial disclosure*: App should identify funding source, company, or publisher
- *Advertising policy:* App should distinguish advertising and paid-service content from editorial content

**Table 1:** Usability heuristics for user interface design.

Heuristic^a,b^	Definition	Questionnaire items
Visibility	The system should always keep users informed about what is going on, through appropriate feedback within a reasonable amount of time	Does every screen begin with a title or header? It is obvious to the user what is going on? Is the font large enough?
Match between system and real world	The system should speak the users’ language, with words, phrases, and concepts familiar to the user, rather than system-oriented terms. Follow real-world conventions, making information appear in a natural and logical order	Are menu choices and information ordered in a logical way? Do related and interdependent information appear together? Is language clear and concise (terminology familiar to users)?
Consistency	Users should not have to wonder whether different words, situations, or actions mean the same thing. Follow platform conventions	Does the app use a minimal number of colors (ie, color consistency)? Is there a consistent design scheme across the app? Do online instructions/information appear in a consistent location across screens?
User control and freedom	Users often choose system functions by mistake and will need a clearly marked emergency exit to leave an unwanted screen without having to go through an extended dialogue. Support undo and redo actions	Is there navigation on the homepage of the app? Can users easily reverse their actions? Is the app explore-able and easy to navigate?
Error prevention	Even better than good error messages is a careful design that prevents a problem from occurring in the first place. Either eliminate error-prone conditions or check for them and present users with a confirmation option before they commit to an action	Are menu choices logical, distinctive, and mutually exclusive? Are buttons/commands placed a good distance from one another? Does the system prevent users from making errors whenever possible?
Recognition rather than recall	Minimize the users’ memory load by making objects, actions, and options visible. The user should not have to remember information from one part of the dialogue to another. Instructions for use of the system should be visible or easily retrievable whenever appropriate	Are instructions visible? Is it obvious what is clickable? Does the app require high levels of concentration?
Flexibility and efficiency of use	Accelerators—unseen by the novice user—may often speed up the interaction for the expert user such that the system can cater to both inexperienced and experienced users. Allow users to tailor frequent actions	Does the app provide function keys for high-frequency commands? Does the app allow for customization (eg, settings, search)? Does the app provide customization for frequency users (eg, log in, saves data)?
Aesthetic and minimalist design	Dialogues should not contain information that is irrelevant or rarely needed. Every extra unit of information in a dialogue competes with and diminishes the visibility of relevant information	Is the layout clearly designed avoiding visual noise? Does the use of images and multimedia content add value? Are images well sized and is the resolution appropriate?
Error recovery	Error messages should be expressed in plain language (no codes), precisely indicate the problem, and constructively suggest a solution	Are there error messages? Is sound, images, or haptics used to signal an error? Are error messages worded so the user understands the problem and what to do next?
Help and documentation	Ideally, the system can be used without documentation, but in the case of questions or confusion, it’s important to provide help and documentation. Any such information should be easy to search, focused on the user’s needs, list concrete steps to be carried out, and not be too lengthy	Are there instructions/help/documentation? Are navigation and instructions easy to find? Are navigation and instructions procedural (how do I use the app)?

^a^Heuristics are not mutually exclusive.

^b^All questionnaire items ranked on the following scale: 1=cosmetic problem only, 2=minor usability problem, 3=major usability problem, and 4=catastrophic usability problem.

## Results

### Content Evaluation

#### Domain 1

Of the 9 evaluated apps, 7 contained no indication that they were supported by science. Of the 2 remaining apps, 1 somewhat met 1 criterion because it included external links to national organizations, though it did not specify whether its content was based on the linked guidelines. The final app almost met the criteria for this domain. It was developed by a company known for conducting research, though it contained no scientific references.

#### Domain 2

Of the 9 apps, 5 fully met this domain’s criteria. Of the remaining 4 apps, 2 only partially met domain 2 criteria—specifically, the criterion for continued engagement (allows users to record and utilize their breathing measurements). However, this app failed to provide instruction on how to use its skill-building content. The 2 remaining apps did not meet any of this domain’s criteria.

#### Domain 3

Of the 9 apps, 3 contained at least one evidence-based stress management strategy and at least one structure. Of the remaining 6 apps, 3 did not have at least one strategy and structure. Of the other 3 apps, 1 did not contain any evidence-based strategies, but somewhat included 2 potential, evidence-based structures through a breathing measure. Given that no empirical evidence was provided, we were unable to establish that these structures were evidence-based. The 2 remaining apps partially contained 2 strategies. One of the apps recommended seeking social support but did not provide tools or resources to do so; the other app contained breathing exercises, but we were unable to establish if these were consistent with diaphragmatic breathing. Of the 2 remaining apps, 1 did not contain any evidence-based structures and 1 app somewhat seemed to contain all 3 structures. Again, however, we were unable to establish that these structures were evidence-based. Among the apps that had at least one evidence-based stress management strategy, the most common strategies were meditation and mindfulness, diaphragmatic breathing, and seeking social support. The most common evidence-based structures were self-monitoring and assessment.

#### Domain 4

Of the 9 apps, 2 did not meet any of this domain’s 8 criteria, and none met all criteria. Some of the apps were predominantly educational and did not collect user data; thus, we did not expect these apps to meet the confidentiality and privacy criteria. Only 1 app met 5 criteria, but it, along with all the other apps, failed to meet the references and advertising policy criteria. However, the latter unlikely applied to this app given that it contained no advertisements. The most commonly met criteria were presentation of contact details and indication that app content intended to complement rather than replace professional medical care.

### Usability Heuristics Evaluation

Usability varied across the apps (Table 2). Of the 10 usability heuristics, half were critically violated by a majority of apps—visibility, match between system and real world, error prevention, recognition, and help and documentation. We will focus our discussion on these 5 (see definitions in Table 1).

Visibility was problematic in 8 of the 9 apps. In particular, some apps had a complex navigation structure, including a lack of headers and feedback, leading to feeling lost in the app. Moreover, 4 apps utilized a small font size, problematic for many cancer survivors over the age of 50 years.

A mismatch between the system and the real world was another usability issue, problematic in 7 apps. Examples included the use of technical jargon, a disorganized menu, and use of advanced yoga terms not obvious in meaning to novice users.

A third usability heuristic was error prevention. Of the 9 apps, 8 contained issues related to disorganized content or functions, or buttons too close together. Furthermore, some apps that required health information (eg, blood pressure), or self-report of medication practices, did not allow users to edit information they had entered or made it very difficult to enter information (due to font size or entry fields).

All 9 apps contained issues related to recognition rather than recall. Instructions were often hard to find (an issue related to 3 other usability heuristics—visibility, help, and documentation). Some apps had so much functionality that they were overwhelming to first-time users. This led to difficulty in navigation, how to use specific functionality (eg, videos, information trackers), and even how to figure out the app’s purpose. There were 2 apps that did offer a tutorial for first-time users, and this feature mitigated some of these recognition or recall issues.

Finally, 8 of the 9 apps contained help and documentation issues. Similar to recognition issues, many of these problems stemmed from a lack of, or difficult-to-find, instructions. Furthermore, developer contact information was often unavailable, making it difficult to request help or report an issue.

### Grade-Level Readability Evaluation

Table 3 shows the results from the grade-level readability evaluation. The grade-level readability results showed that 7 of the 8 apps contained content at the ninth-grade reading level or higher. Only 1 app had a reading level below eighth grade, whereas 1 did not have enough text to calculate a readability score.

### Cultural Sensitivity

Of the 9 apps evaluated, none met any of the CSC criteria. One of the apps was predominantly intended for tracking symptoms and thus contained minimal text, making it difficult to evaluate cultural sensitivity (same app for which we could not calculate a readability score).

**Table 2:** Average usability heuristic scores across apps.

App^a^	Heuristic^b^										Mean (SD)^c^	
	V	M	CS	CT	EP	R	F	A	ER	H		
1	3.00	3.50	1.17	2.17	2.67	3.33	2.83	2.00	0.67	2.83		2.42 (0.92)
2	1.50	0	0	0.17	0.67	0.67	2.00	0	1.83	0.17		0.70 (0.79)
3	3.00	3.00	2.20	3.00	3.00	3.20	2.60	3.20	1.00	3.00		2.72 (0.67)
4	1.80	2.00	2.00	0.80	1.40	2.40	0.80	2.00	0	2.40		1.56 (0.79)
5	3.50	3.50	3.00	3.50	3.50	3.33	2.50	2.33	3.17	3.83		3.22 (0.48)
6	1.83	2.17	2.00	1.83	2.50	2.33	1.50	2.00	2.67	2.83		2.17 (0.42)
7	0.80	1.20	0	0	0.40	0.80	0.60	0	0	1.20		0.50 (0.49)
8	2.20	3.20	3.00	3.20	2.80	3.00	1.00	0	2.40	2.40		2.32 (1.05)
9	0.50	0.50	2.50	1.75	1.25	2.25	0.50	1.25	1.50	1.50		1.35 (0.71)
Mean (SD)^c^	2.01 (1.02)	2.12 (1.31)	1.76 (1.14)	1.82 (1.29)	2.02 (1.11)	2.37 (1.02)	1.59 (0.92)	1.42 (1.18)	1.47 (1.15)	2.24 (1.11)		—

^a^Apps: 1=COPD Disease (Droid Clinic, United States); 2=Lung+ Pioneering Healthcare (Roche, Indianapolis, Indiana, United States), 3=COPD (Health Tips, United States), 4=Pranayama Free (Sagaara, Ann Arbor, Michigan, United States), 5=Breathcount (Segfoltas, Kaunas, Lithuania), 6=Asthma Tracker and Log (Roving Reptiles Software, Castle Rock, Colorado, United States), 7=My Breathfree (Cipla Digital. Sussex, England), 8=7Pranayama—Yoga Breath Calm (Pixel Point Technology, Jaipur, India), and 9=Loving Meditations—Bring Calm To Cancer (Loving Meditations, New York, New York, United States).

^b^V: visibility; M: match between system and real world; CS: consistency; CT: user control and freedom; EP: error prevention; R: recognition rather than recall; F: flexibility and efficiency of use; A: aesthetic and minimalist design; ER: error recovery; H: help and documentation.

^c^Higher score indicated a greater frequency, impact, and persistence of usability issue. All questionnaire items were ranked on the following scale: 1=cosmetic problem only, 2=minor usability problem, 3=major usability problem, and 4=catastrophic usability problem. Mean and SD were calculated based on average score (1-4) across all heuristics and within each separate heuristic.

**Table 3:** Grade-level reading scores. COPD: chronic obstructive pulmonary disease.

App name	Grade-level reading score
My Breathefree	7.4
COPD Disease	12.6
Lung+ Pioneering Healthcare	9.4
COPD	9.2
Pranayama Free	12.5
Asthma Tracker & Log	9.1
7pranayama—Yoga Breath Calm	10.5
Loving Meditations—Bring Calm to Cancer	12.8
Breathcount	Unable to calculate

## Discussion

### Principal Findings

Of the 9 apps evaluated, 3 focused on providing support for individuals with asthma (My Breathefree, Breathcount, Asthma Tracker and Log), 2 aimed to support individuals with COPD (COPD Disease, COPD), 2 provided general relaxation and breathing training (Pranayama Free, 7pranayama—Yoga Breath Calm), and 2 contained videos and exercises to support individuals with lung cancer (Loving Meditations—Bring Calm to Cancer, Lung+ Pioneering Healthcare).

Though all 9 apps were marketed as breathing management and stress reduction, only 3 met the criteria for having both an evidence-based stress management strategy and an evidence-based stress management structure (ie, assessment, self-monitoring, or psychoeducation features; see Textbox 2). For example, Loving Meditations —Bring Calm to Cancer included 5 evidence-based stress management strategies that we assessed—meditation or mindfulness, diaphragmatic breathing, cognitive restructuring, visualization and imagery, and active coping or behavioral activation.

None of the apps fully met the criteria for providing scientific evidence to support claims about information within, or the efficacy of, their app. The 2 apps that somewhat met criteria for having scientific evidence—Lung+ Pioneering Healthcare and COPD—both contained content linked to a corporation (eg, Roche), and neither referenced peer-reviewed literature or other scientific evidence. In addition, most apps either met or partially met the criteria for being interactive or engaging, and over half of these incorporated a skill-building component. For example, in addition to offering breathing exercises and general lung education, Lung+ Pioneering Healthcare featured a multistage, interactive saxophone player breathing game that challenged players to blow rhythmically into their mobile microphones to the tune of jazz music.

Transparency was variable across the apps with Lung+ Pioneering Healthcare meeting the most criteria (5 of 8). The average grade-level readability in our review was 10th grade, which is 2 grades above the acceptable level [23]. Many of the apps had usability challenges identified as critical violations. Finally, none of the apps met any of the criteria for being culturally sensitive to African Americans, who are more likely to experience lung cancer mortality [29] and may have earlier onsets of COPD [25].

### Limitations

This review focused on evaluating apps for the most common, evidence-based stress management techniques (ie, meditation and mindfulness, diaphragmatic breathing, and seeking social support). Therefore, less-common evidence-based stress management strategies may be used but were excluded in our review.

### Comparison With Prior Work

Compared with Coulon and colleagues’ findings [20], the apps reviewed were less likely to include at least one evidence-based stress management strategy. However, when apps employed these strategies (eg, meditation), they were similar to those found in the previous review. Our findings regarding apps’ scientific merits were comparable to those of Coulon and colleagues [20], but their review yielded more apps with scientific references (33% vs our 0%).

Regarding transparency, our review produced results consistent with those of Coulon and colleagues [20], but criteria most and least often met differed. Specifically, apps in both reviews were likely to provide contact information, but other criteria (eg, advertising policy) were satisfied less often in our review. Our assessment of apps for inclusion of skill-building instructions and opportunities for continued engagement demonstrated that most apps met or partially met these criteria. Although Coulon and colleagues did not assess cultural sensitivity or readability, our findings regarding an absence of cultural sensitivity and low readability were consistent with other prior work [28,30,31].

Finally, our review included a comprehensive heuristic evaluation to determine usability, which was more in-depth than the review by Coulon and colleagues [20]. Therefore, we identified critical design flaws that may affect users’ ease of use.

On the basis of our evaluation, we make 5 key recommendations for improving the quality of commercially available apps aimed at adults with a COPD or lung cancer history.

1. *Institute more stringent regulation of apps for health*. Apps that make therapeutic claims or present health-related information should be required to cite scientific evidence to substantiate their claims or information. These apps should also contain prominent disclaimers about the outcomes a user should expect, particularly when claims are made about benefiting users’ health. Currently, no federal regulatory standards govern the production of commercially available, health-related apps (eg, those that provide health education) [32]. The Food and Drug Administration, Google, and Apple provide some guidance to app developers in their review guidelines for medical and health-related apps. For example, Section 1.4.1 of Apple’s App Store Review Guidelines states: “Medical apps that provide inaccurate data or information, or that could be used for diagnosing or treating patients may be reviewed with greater scrutiny. Apps must clearly disclose data and methodology to support accuracy claims relating to health measurements, and if the level of accuracy or methodology cannot be validated, we will reject your app.” [33]. Language in Google’s Developer Policy Center simply states that it does not allow “apps that contain false or misleading information or claims, including in the description, title, icon, and screenshots” such as “apps that feature medical or health-related functionalities that are misleading or potentially harmful.” [34]. Beyond this brief language, however, neither Apple nor Google provide any insight into their review process, such as who reviews the apps they sell (eg, MD-degree holders hired by Apple). Moreover, no information exists for how nonmedical health apps (such as those we evaluated) are reviewed. We recommend that these organizations embrace a rigorous and transparent regulatory process to evaluate the health content within an app. One set of digital health reviewer principles is published by the Health On the Net Foundation [35]. For a more in-depth regulatory process, Google, Apple, or other independent distributors of health-related apps could partner with the clinical and scientific communities to review these apps (similar to an expert or peer-review process). Though the peer review would be voluntary, it could ensure the quality of health-related apps. Furthermore, these organizations should assure greater scrutiny of app descriptions to ensure that developers are accurately reporting the contents of an app, and they should also denote whether or not the app is evidence-based. This could give the user a quick way to determine the legitimacy and scientific merit of the app.
2. *Use evidence-based frameworks and participatory design processes for app design.* Although both Apple and Google provide guidance for app development largely based on industry standards, the apps evaluated were highly variable in the extent to which they executed these guidelines. Usability standards should be updated for mobile devices because most current guidelines were built with desktop layouts in mind. More specifically, Yáñez Gómez and colleagues [22] recalibrated Nielsen’s [21] heuristics for mobile devices, which have different usability challenges than desktop computers (eg, screen-size limitations). One recommendation is to not only make apps follow platform conventions but also be consistent across mobile orientations (ie, whether the device is vertical or horizontal) [22]. Conforming to specific guidelines such as these may increase uniformity across apps while preserving the developers’ ability to create unique app designs.
  In addition to normal user testing, which is part of a traditional app development cycle, Owens [36] has recommended implementing a community-based participatory (CBPR) design process. There are 8 CBPR principles that encourage active partnership between developers and the target population [37]. Such collaborations can enhance developers’ abilities to make optimal decisions, from inception to dissemination, by identifying users’ content and usability needs and by jointly creating viable solutions [37]. For example, in Owens’s study [38], CBPR was implemented in a computer-based cancer education program for African American men. Small-group reviews, storyboarding, and user testing ensured the program was culturally appropriate, easy to understand, and usable by the target population [36,38]. Though integrating CBPR principles into the app design lifecycle can be more time-consuming, the cultural and contextual relevance and usability of interventions is increased, thereby increasing the likelihood that the app will contribute to positive health outcomes [36,39,40].
3. *Use culturally sensitive language and images in health-related apps.* Although lung cancer mortality is more common among African Americans [26] and this racial group may have earlier onsets of COPD [27], no apps evaluated were rated as culturally sensitive toward this population. Evidence suggest that ethnic minorities have distinct cultural beliefs that affect their beliefs about chronic lung disease, including how they engage in care [41,42]. General cancer-related studies have also demonstrated a desire among ethnic minorities for culturally relevant health information [43,44]. However, many print and online education materials are not culturally sensitive [25,28]. To ensure that apps are culturally sensitive, developers should consider using an existing grading tool or checklist such as the Cultural Sensitivity Assessment Tool (CSAT) for African Americans or CSC [28,45]. These tools are attentive to details in content and imagery that may be overlooked in a general design lifecycle. Implementing a CBPR-focused app design process with a representative sample of the target population (as mentioned above) offers an effective means for ensuring all CSAT or CSC recommendations are implemented.
4. *Ensure that apps are written in plain language.* One objective of the US Department of Health and Human Services’ Healthy People 2020 initiative is to improve health literacy [46]. Health literacy is defined as the “degree to which individuals have the capacity to obtain, process, and understand basic health information and services needed to make appropriate health decisions.” [46,47]. Individuals without an adequate understanding of health information are less likely to adopt healthy behaviors, leading to poorer health outcomes [48]. There are multiple strategies recommended for enhancing health literacy [48]. One strategy is to ensure that health information is written in plain language—ie, in a manner that is easy to understand for the intended audience [49]. The Plain Writing Act of 2010 (H.R. 946/Public Law 111-274) mandates that all federal agencies adhere to strict plain-language standards for government-provided information regarding benefits or compliance with requirements set forth by the government, including health information [50]. One primary measure of plain language is grade-level readability [51]. Although there is some debate regarding the optimal reading level for health information, the standard has generally been sixth to eighth grade [51]. Readability levels can be determined using one of many readability scales, as we used in our review [24]. Many scales have been digitized which enables users to quickly generate readability scores. We recognize that this measurement does not always translate to the easy comprehension of text [52], but it represents an important step toward greater accessibility of health-related information.
5. *Follow evidence* **.** Our target user group consisted of lung-cancer survivors, typically aged 50 years and older. Apps designed for this population need to use larger font (for better visibility), employ nontechnical language (to improve the user or real world match), have features that mitigate the ability to introduce errors when entering information (including allowing for easy editing), accommodate users’ working memory limitations (relying on recognition rather than recall), and provide ample help if or when users encounter problems (help and documentation). These recommendations mirror evidence-based design recommendations for older users [53]. Following usability guidelines will not only ensure ease of use but also increase acceptance and adoption of mobile app technology.

### Conclusions

Few mobile apps exist for promoting mindfulness-based strategies among adults with a chronic lung disease. Among those available, few meet the criteria for the 4 content evaluation domains (evidence of science, scientific strategy and engagement, evidence-based stress management strategies and structures, and transparency). Although the usability of apps reviewed varied greatly, most had design flaws that may compromise their helpfulness to populations with low technology usage or self-efficacy or limited experience with apps. In addition, the app content was not culturally sensitive or written for audiences with lower reading levels. To enhance the accessibility of evidence-based, commercially available apps for promoting mindfulness-based strategies among adults with a chronic lung disease, we outlined 5 key recommendations. Future research should assess the feasibility and efficacy of implementing these recommended processes within an app development lifecycle. Additionally, future app reviews should also include an assessment of apps for potentially harmful strategies.

## Abbreviations

A: aesthetic and minimalist design
COPD: chronic obstructive pulmonary disease
CS: consistency
CSAT: Cultural Sensitivity Assessment Tool
CSC: Cultural Sensitivity Checklist
CT: user control and freedom
EP: error prevention
ER: error recovery
F: flexibility and efficiency of use
H: help and documentation
M: match between system and real world
MBSR: mindfulness-based stress reduction
R: recognition rather than recall
V: visibility
